# Risk stratification for repeat stone surgery: the role of stone composition

**DOI:** 10.1007/s00345-025-05573-w

**Published:** 2025-04-01

**Authors:** Sagi A. Shpitzer, Igal Shpunt, Nadav Loebl, Leor Perl, Dmitry Enikeev, Abd E. Darawsha, Yaron Ehrlich, David Lifshitz

**Affiliations:** 1 Institute of Urology, Rabin Medical Center, Petah Tikva, Israel; 2 Faculty of Medical and Health Sciences, Tel Aviv University, Tel Aviv, Israel; 3 Artificial Intelligence Center, Beilinson Medical Center Innovation, Rabin Medical Center, Beilinson Campus, Petah Tikva, Israel; 4 Faculty of Computer Science, Reichman University, Herzliya, Israel; 5 Department of Cardiology, Rabin Medical Center, Beilinson Campus, Petah Tikva, Israel; 6 Urology Department, Medical University of Vienna, Vienna, Austria

**Keywords:** Percutaneous nephrolithotomy, Stone composition, Ureteroscopy, Urolithiasis

## Abstract

**Purpose:**

Kidney stones have a recurrence risk of 30–50% within five years, with surgical recurrence often being the most clinically significant and burdensome. Stone composition results obtained after surgery are readily available and typically precede metabolic evaluation. However, only few, relatively small studies, correlated stone composition with surgical reccurence. This study aims to determine whether stone composition alone can reliably predict recurrent stone surgery, offering insights into personalized management strategies.

**Methods:**

A retrospective analysis of surgically treated patients with an available stone composition analysis performed between 2013 and 2020 in a large healthcare provider database. Data were analyzed for up to 5 years from the initial surgery.

**Results:**

8,561 patients underwent surgical procedures for stones and 2,097 (24.5%) had repeat surgery within 5 years. Compared to calcium oxalate stone formers, individuals forming uric acid, calcium phosphate, infection, brushite, and cystine stones were 1.5, 1.5, 1.87, 2.64, and 2.71 times more likely, respectively, to undergo a second stone surgery (*p* < 0.001). The median time for repeat stone surgery in cystine and infection stone formers was significantly shorter compared to calcium oxalate (*p* < 0.01).

**Conclusions:**

Stone composition significantly affects the 5-year surgical recurrence rates and the median time to recurrence, with non-calcium oxalate stone formers at a higher risk for recurrence. Sharing this information with patients may improve compliance with preventive strategies, including comprehensive metabolic evaluation and adherence to preventive therapy. Emphasizing this risk may help prioritize proactive management in high-risk patients.

**Supplementary Information:**

The online version contains supplementary material available at 10.1007/s00345-025-05573-w.

## Introduction

Global trends indicate a notable rise in the occurrence of kidney stones over recent decades. Consequently, healthcare spending has escalated, surpassing $2 billion in the USA alone [1]. Epidemiological data reveal a lifetime risk of stone formation ranging from 5 to 10%, with recurrence rates up to 50% within 5 years and reaching 70–80% within a decade [2–5]. Reducing stone recurrence may decrease the burden of kidney stone disease for both individual patients and healthcare systems. Metabolic evaluation and preventive measures represent pivotal strategies in this endeavor. While guidelines from both the American Urological Association and the European Association of Urology advocate for 24-hour urine collection in high-risk individuals, real-world data indicate low rates of metabolic evaluations among such patients. For instance, in a large population-based study of 28,836 high-risk patients, only 7.4% underwent metabolic evaluations [6]. A higher rate of metabolic evaluations (39%) was observed among patients who have undergone stone surgery [7].

Patient compliance with long-term preventive therapy remains suboptimal. We have recently shown that approximately half of patients with metabolic abnormalities prescribed secondary chemoprevention with hydrochlorothiazide and alkali citrate failed to adhere to the prescribed regimen [8]. Given the widespread prevalence and variable course of nephrolithiasis, preventive strategies should prioritize patients with a relatively high risk for recurrent stones. Those who have undergone stone surgery may represent a more motivated cohort to undergo complete metabolic evaluation and adhere to preventive therapy. Further stratification of risk factors for recurrence within this group can assist in counseling patients at heightened risk for repeat surgery. Few studies have assessed the relative risk and timing of repeat stone surgery by stone composition [9–11]. Therefore, the purpose of the current study was to investigate in a large patient cohort the relative risk for repeat surgery of non-calcium oxalate stone formers compared to calcium oxalate stone formers.

## Materials and methods

### Setting and patients

This retrospective longitudinal study was approved by the institutional review board of Clalit Health Services (CHS; 0136 − 22 RMC). CHS is the largest health maintenance organization (HMO) in Israel. It includes 14 hospitals across the country and provides primary, secondary, and tertiary medical services to almost 5 million people. The CHS database provides access to a wide range of clinical data, as it gathers information from various live logistical, pharmaceutical and medical sources.

The study cohort included all adult patients who had been surgically treated for nephrolithiasis and had results of a stone analysis between 2013 and 2020. The starting year was chosen because at that time Fourier Transform Infrared (FTIR) spectroscopy analysis completely replaced the obsolete chemical analysis technique. Data collection was concluded in August 2023, allowing for a follow-up of at least 2.5 years for each patient.

### Stone composition analyses

All stone composition analyses were performed at a single centralized laboratory. Stone composition was determined using the ALPHA FTIR spectrometer (Bruker, Karlsruhe, Germany) and compared with Bruker’s BLG 1&2 spectral libraries for stone compositions.

Patients were grouped according to stone composition which was primarily defined according to the most prevalent stone type on analysis. Exceptions included stones containing > 10% of either cystine or brushite, and infection stones, which were defined as predominantly struvite or a combination of carbapatite with > 10% struvite as previously described [12]. All other mineral phases of predominantly calcium phosphate stones were defined as calcium phosphate, as described by Daudon et al. [10]. Calcium oxalate dihydrate and monohydrate were grouped together. If a patient had multiple stone analyses during the study period, the first stone analysis was used.

### Data collection, definitions and outcomes

Demographic data collected included sex and age at first stone analysis or first recorded surgery. Clinical data included body mass index (BMI), estimated glomerular filtration rate (eGFR) according to the Chronic Kidney Disease Epidemiology Collaboration (CKD-EPI) creatinine equation, comorbidities and surgery for nephrolithiasis.

Comorbidities were defined according to the International Classification of Diseases, Tenth Revision (ICD-10) diagnoses in the patients’ records and were chosen according to previous studies which have shown a correlation between these conditions and stone disease [11]. Data on comorbidities included diabetes, hypertension, gout, ischemic heart disease (IHD), inflammatory bowel disease (IBD) and chronic kidney disease (CKD). The total number of these comorbidities per patient was calculated. Obesity was defined as BMI > 30 kg/m^2^. Billing information regarding surgery was fully available for all patients undergoing surgery in CHS hospitals. Surgery for the treatment of nephrolithiasis was defined according to relevant ICD 9-CM-Volume 3 procedure codes (supplementary material).

The primary endpoint was recurrent stone surgery, defined as any surgical procedure for nephrolithiasis occurring more than 6 months following the first recorded stone surgery and within 5 years. The five-year cutoff is a widely accepted time frame for evaluating recurrence rates.

### Statistical analysis

Continuous variables were summarized using their average and standard deviation, and categorical variables were given with their number and percentages. T-test, analysis of variance (ANOVA) and Welch’s test were used to compare continuous variables, and chi-squared test was used for categorical variables. Kaplan Meier curves were used to demonstrate surgical stone recurrence. Statistical analysis was performed using SPSS Statistic Version 28.0 (IBM Corp, Armonk, NY, USA), with *p* value < 0.05 considered statistically significant. Python 3.9 was also used for data analysis and computational tasks.

## Results

A total of 8,561 patients met the study eligibility criteria. The cohort’s demographic and clinical data are presented in Table 1. Initial surgery was extracorporeal shockwave lithotripsy (ESWL), percutaneous nephrolithotomy (PCNL) and ureteroscopy in 1,498 (17.5%), 790 (9.2%) and 6,273 patients (73.3%) respectively.

**Table 1:** Demographic and healthcare characteristics of the study population by stone composition

	Stone composition						*p* value
	Calcium Oxalate *N* = 7000	Uric Acid *N* = 1004	Calcium Phosphate *N* = 140	Infection *N* = 318	Brushite *N* = 45	Cystine *N* = 54	
Age^a^ (years), mean ± SD	50.9 ± 14.9	60.3 ± 13.2	47.73 ± 16	53.24 ± 18.8	42.74 ± 16.4	37.9 ± 18.8	**< 0.001**
Males, n (%)	5,300 (75.7%)	692 (68.9%)	61 (43.6%)	124 (39%)	34 (75.6%)	30 (55.6%)	**< 0.001**
BMI (kg/m^2^), mean ± SD	28.1 ± 5.2	31.2 ± 6.2	26.1 ± 4.6	28.4 ± 5.8	27.8 ± 6.8	28.4 ± 7.1	**< 0.001**
eGFR (CKD-EPI, ml/min), mean ± SD	89.3 ± 21.8	69.6 ± 22.7	92.9 ± 29.6	88.1 ± 29.9	98.4 ± 19.4	80 ± 25.9	**< 0.001**
Comorbidities, n (%)							
Chronic kidney disease	940 (13.4%)	439 (43.7%)	25 (17.8%)	76 (23.9%)	7 (15.5%)	24 (44.4%)	**< 0.001**
Obesity	2,147 (30.7%)	523 (52.1%)	27 (19.3%)	105 (33%)	15 (33.3%)	15 (27.8%)	**< 0.001**
Diabetes	2,182 (31.2%)	614 (61.1%)	25 (17.9%)	102 (32.1%)	12 (26.7%)	15 (27.8%)	**< 0.001**
Hypertension	3,248 (46.4%)	789 (78.6%)	58 (41.4%)	157 (49.8%)	20 (44.4%)	22 (40.7%)	**< 0.001**
Gout	711 (10.2%)	225 (22.4%)	5 (3.6%)	27 (8.5%)	5 (11.1%)	6 (11.1%)	**< 0.001**
Inflammatory bowel disease	164 (2.3%)	22 (2.2%)	2 (1.4%)	1 (0.3%)	1 (2.2%)	1 (1.8%)	0.28
Ischemic heart disease	1,422 (20.3%)	375 (37.3%)	17 (12.1%)	67 (21.1%)	5 (11.1%)	8 (14.8%)	**< 0.001**
Comorbidity sum, mean ± SD	1.26 ± 1.3	2.47 ± 1.3	1.01 ± 1.1	1.38 ± 1.3	1.13 ± 1.2	1.43 ± 1.3	**< 0.001**
Ureteroscopy as 1st procedure n (%)	5,181 (74%)	741 (73.8%)	100 (71.4%)	191 (60.1%)	30 (66.7%)	30 (55.6%)	**< 0.001**

^a^ Age at first stone analysis or first recorded surgery

Abbreviations: BMI, body mass index; eGFR, estimated glomerular filtration rate; CKD-EPI, Chronic Kidney Disease Epidemiology Collaboration; SD, standard deviation

Patients with uric acid stones were older than those in all other groups (average age 60.3 ± 13.2 years, *p* < 0.001) while cystine stone formers were younger (average age 37.9 ± 18.8 years, *p* < 0.001). In all stone composition groups most patients were male except for the infection stones and calcium phosphate stones groups, which comprised 61% and 56% female patients, respectively (*p* < 0.001). Estimated GFR was significantly lower in the uric acid group and in the cystine group compared to all other groups (69.6 ± 22.7 ml/min and 80 ± 25.9 ml/min, respectively). Patients with uric acid stones had a significantly higher average number of comorbidities than those in the other groups (2.47 ± 1.3 comorbidities/patient) and, except for IBD, the prevalence of all evaluated comorbidities was higher in this group compared to the other groups.

Complete 5-year follow up was available for 65.8% of the cohort with the remaining 34.2% having a follow up of between 2.5 and 5 years. 11,982 surgical procedures for the treatment of stones were recorded among the 8,561 patients in our cohort. Repeat surgery within a 5-year period was reported for 2,097 patients (24.5%) (Table 2). During the follow up period 1,613 patients (76.9%) had a single surgical recurrence, 370 patients (17.6%) had 2 recurrences and 114 patients (5.4%) had 3 or more surgical recurrences in a 5-year period.

**Table 2:** Five-year surgical stone recurrence by stone composition

	Stone composition						
	Calcium Oxalate *N* = 7000	Uric Acid *N* = 1004	Calcium Phosphate *N* = 140	Infection *N* = 318	Brushite *N* = 45	Cystine *N* = 54	Total *N* = 8561
**Recurrent stone surgery n**,** (%)**	1533 (21.9%)	330 (32.9%)	46 (32.9%)	130 (40.9%)	26 (57.8%)	32 (59.3%)	2097 (24.5%)
Relative Risk (RR) (95% confidence interval)	1	1.5 (1.36–1.66)	1.5 (1.18–1.91)	1.87 (1.62–2.15)	2.64 (2.05–3.4)	2.71 (2.16–3.39)	
*p* value	NA	< 0.0001	0.002	< 0.0001	< 0.0001	< 0.0001	

**Fig. 1:**
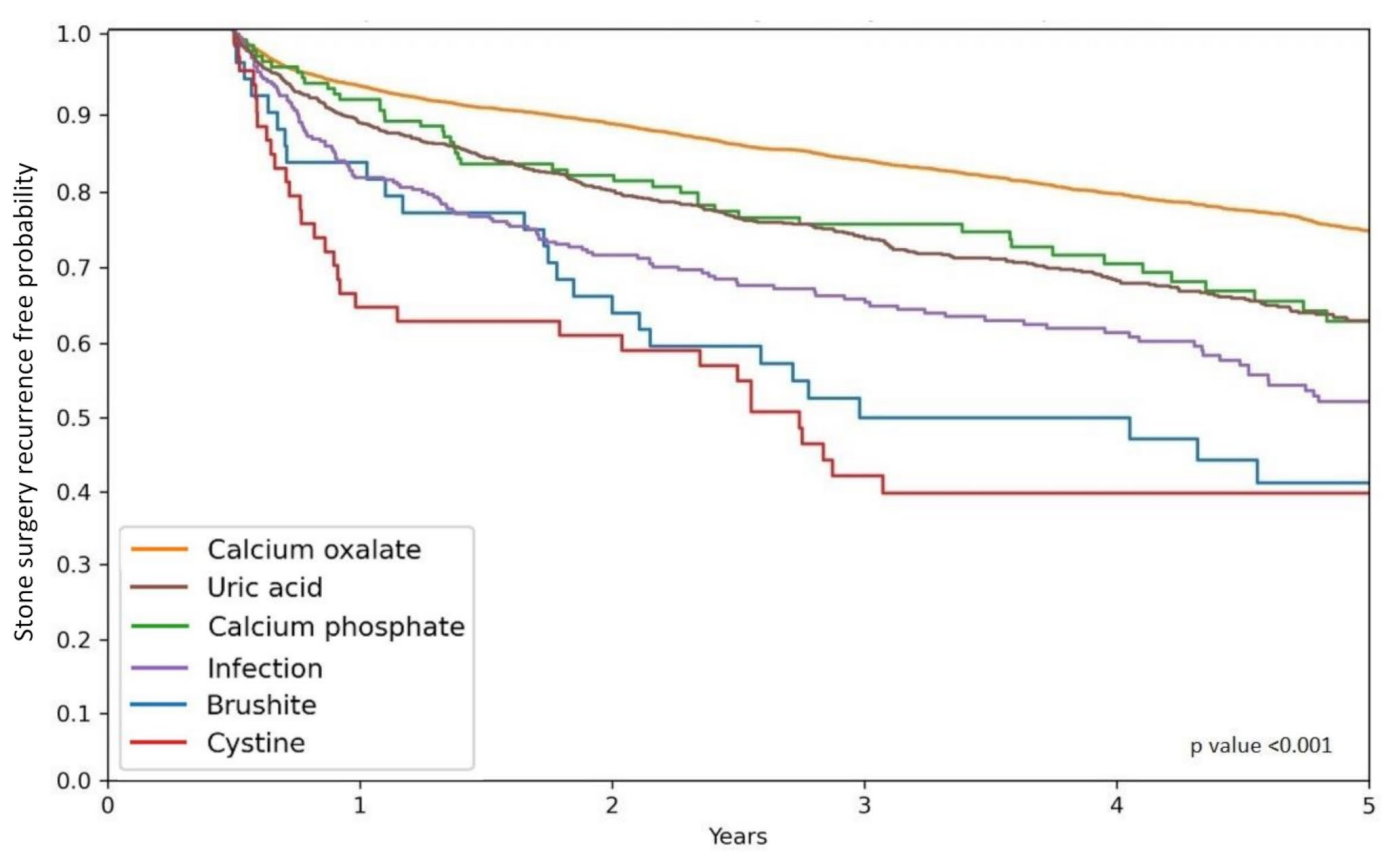
Kaplan-Meier curves for stone recurrence by stone composition

Calcium oxalate stone formers (SF) had the lowest 5-year recurrent stone surgery rate (21.9%), whereas cystine SF had the highest recurrent stone surgery rate (59.3%), and therefore were 2.71 times more likely to undergo surgery in comparison to those in the calcium oxalate group. Patients with uric acid, calcium phosphate, infection and brushite stones were 1.5, 1.5, 1.87 and 2.64 times more likely to experience recurrent stone surgery within 5 years in comparison to calcium oxalate SF. Figure 1 shows the Kaplan-Meier survival analysis for recurrent stone surgery by stone type.

The median time to recurrent stone surgery among the 2,097 patients who underwent recurrent surgery during the 5-year period from first surgery was 561 days. Patients with cystine and infection stones exhibited significantly shorter median times to recurrent surgery compared to those with calcium oxalate stones, with durations of 321 and 421 days respectively, versus 602 days (*p* < 0.01). Patients with uric acid stones showed a trend toward statistical significance with 534 days versus 602 days for calcium oxalate stones (*p* = 0.053). No notable difference was observed for patients with brushite or calcium phosphate stones (634 and 505 days respectively, *p* > 0.05).

## Discussion

Recurrent stone surgery is arguably the most critical type of stone recurrence. To the best of our knowledge, this study is the largest population-based study focusing on the risk of recurrent stone surgery based solely on stone analysis. As stone composition is readily available for most patients undergoing endoscopic procedures, it can be used to improve compliance with comprehensive metabolic evaluations, especially in patients at higher risk of recurrence.

The largest published longitudinal study to date, which analyzed the predictors of recurrent symptomatic nephrolithiasis (the ROKS nomogram), included only first-time SF, around two-thirds of whom had not undergone surgical intervention. Moreover, stone composition data were available for only 49% of the cohort [11]. The ROKS nomogram incorporated any known uric acid composition as a predictor for increased recurrence, while calcium oxalate stones tended to recur less frequently. The revised ROKs study added brushite and struvite as risk factors for symptomatic stone events [9]. In the current study we have further explored the impact of various stone compositions on recurrent stone surgery.

The overall 5-year surgical recurrence in our cohort was 24.5%. The recurrence rate among patients with calcium oxalate stones was 21.9%, close to the overall recurrence rate, as expected for a group comprising the majority of patients. This patient group was the least likely to undergo recurrent stone surgery and therefore may be categorized as a low-risk group. Patients with uric acid and calcium phosphate stones can be categorized as a medium-risk group with a 5-year surgical recurrence of 32.9% each. The high-risk group includes infection, brushite, and cystine stones with a 5-year surgical recurrence of 40.9%, 57.8%, and 59.3%, respectively (Table 2). This data is particularly meaningful for patients with non-calcium oxalate stones. Compared to individuals forming calcium oxalate stones, those forming uric acid, calcium phosphate, infection, brushite, and cystine stones are 1.5, 1.5, 1.87, 2.64 and 2.71 times more likely, respectively, to require a second stone surgery within 5 years following the initial surgery. These differences among the stone composition groups were highly significant (*p* < 0.001). Moreover, the median time to recurrence in most non-calcium oxalate stone groups was notably shorter compared to calcium oxalate SF. These findings and risk stratification can be easily explained to patients, aiding in the selection of patients for closer follow-up and more comprehensive metabolic evaluation in an attempt to decrease their heightened risk of surgical recurrence. It is important to note that these results reflect the entire cohort irrespective of any general or specific preventive measures that some patients received.

The overall rate of 24.5% of patients undergoing repeat stone surgery within 5 years of the first stone surgery is lower than that reported by Daudon et al. [10] who examined the recurrence rates of urinary calculi by stone composition. In their analysis, 42.7% of 38,274 patients experienced a second stone event. However, stone events were not specified and were not limited to surgical interventions. Furthermore, the time span of recurrence was not specified or limited, and thus may have extended far beyond the 5-year cutoff. Nevertheless, similar to our results, Daudon et al. found that the highest recurrence rate was observed in patients with cystine stones followed by brushite stones, and that patients with calcium oxalate monohydrate (COM) stones had the lowest recurrence rate. Although we did not separate the crystalline phases of calcium oxalate stones in the current study, in a previous study, we have shown that COM is the predominant component of most calcium oxalate stones in Israel [13].

In a similar study to ours, which included 1051 patients from a single center, Li et al. [14] reported an overall stone recurrence rate of 26.7%. However, stone analysis was performed by two separate commercial laboratories, a factor that may have influenced the results, particularly those of calcium phosphate stones that may have been categorized differently. To avoid including staged procedures, repeat surgery was defined by Li et al. [14] as a procedure performed more than 3 months after the initial surgery. We have extended this time limit to 6 months and thus may have avoided some auxiliary procedures following the initial surgery. Although Li et al.’s study captured repeat surgery after the 5-year cutoff, with a median follow-up of 4.7 ± 2.5 years, the surgical recurrence-free survival curves among patients stratified by stone composition were remarkably similar to the ones observed in the current study. Interestingly, when Li et al. added pre- and postoperative stone sizes to the model, calcium phosphate stone composition was found to be an insignificant predictor for recurrence. After adding all other clinical and demographic factors to the model, uric acid stone composition lost its significance regarding recurrence. We reported similar results in a previous study comprising 457 patients who underwent ureteroscopy and percutaneous nephrolithotomy with a minimum follow-up of 2 years [15]. In comparison to calcium oxalate stones, uric acid, struvite and cystine stones were predictors for recurrence in a univariate analysis. A fifth of the patients in the cohort were uric acid SF (21.7%). Symptomatic recurrence occurred in 20.2% of uric acid SF in comparison to 13.4% of calcium oxalate SF (*p* < 0.001). However, in a multivariable analysis, which included demographic data, preoperative stone size and medical prevention, only uric acid remained as an independent predictor (*p* = 0.04). In the current study, we did not analyze the potential predictive value of available clinical and demographic factors. Instead, we chose to focus solely on stone composition, which often precedes a complete metabolic evaluation. We believe that, since our cohort was significantly larger than those in previously published studies and included data from multiple centers, these findings reflect ‘real-world’ results and are of substantial value to patients.

Iremashvili et al. [16] conducted an external validation of the ROKS nomogram, employing more strict criteria focusing solely on patients who had undergone surgical interventions. Their study encompassed 498 first-time SF who had undergone surgical treatment, with a median follow-up of 4.8 years. In this study 17.7% of patients experienced surgical recurrence and analysis revealed three significant risk factors: age, the presence of concurrent asymptomatic stones, and symptomatic stones located in the renal pelvis or lower pole. Patients with uric acid stones were not found to be at higher risk for recurrence, however, only 45% of patients had information on stone composition, with uric acid stones accounting for merely 6% of them.

The current study has some limitations inherent to large de-identified HMO data. Our results may have underestimated the absolute surgical recurrence rate as some of the patients may have undergone surgery in hospitals not connected to the database. However, clearly the choice of provider is not influenced by stone composition and therefore the relative risk for recurrent surgery based on stone composition should not have been affected. Lack of access to imaging results precluded information on preoperative stone burden and its laterality, and additionally as a result of this limitation stone-free status following initial surgery could not be verified in this large anonymized data base. If certain stone compositions tended to present with higher stone burdens their initial stone free rate may have been lower and have necessitated additional surgical procedures. In order to mitigate this limitation as much as possible we used a generous 6-month minimum interval between surgeries as a proxy for stone-free status, assuming most patients who were not stone free after the initial surgery would be retreated in this window and thus would not be counted as a surgical recurrence. Nevertheless, it is reasonable to assume that those few cases operated on for a residual stone at an interval > 6 months will be spread evenly across the various stone compositions.

## Conclusions

Stone composition significantly impacts 5-year recurrent stone surgery rates and the median time to recurrence. Non-calcium oxalate stone formers are at higher risk for repeat stone surgery. Sharing this data with patients can be valuable in counseling them on the importance of metabolic evaluations and the adoption of preventive measures.

## Electronic supplementary material

Below is the link to the electronic supplementary material.

Supplementary Material 1

## Data Availability

The datasets used during the current study are not publicly available since the Clalit Research Institute’s protocols preclude uploading datasets to the internet due to ethical and privacy concerns. Deidentified data may be available for research purposes from the corresponding author upon reasonable request.
