# Supplementary material for: Risk stratification for repeat stone surgery: the role of stone composition
Source: World J Urol. 2025 Apr 1;43(1):203. doi: 10.1007/s00345-025-05573-w (PMC11961492; doi:10.1007/s00345-025-05573-w)
Supplement: Supplementary file 1 — Supplementary Material 1 [file 345_2025_5573_MOESM1_ESM.docx]

**Risk stratification for repeat stone surgery: the role of stone composition**

**World Journal of Urology**

Sagi A. Shpitzer, Igal Shpunt, Nadav Loebl, Leor Perl, Dmitry Enikeev, Abd E. Darawsha, Yaron Ehrlich, *David Lifshitz

*Corresponding author - David Lifshitz - Institute of Urology, Rabin Medical Center, Petah Tikva, Israel & Faculty of Medical and Health Sciences, Tel Aviv University, Tel Aviv, Israel

email: davidlif@clalit.org.il

Supplementary material – ICD 9-CM- Volume 3 codes used to search for surgical procedures for the treatment of urinary stones –

1. 98.51 – Extracorporeal Shock Wave Lithotripsy
2. 55.0 – Percutaneous Nephrolithotomy
3. 56.0, 56.31 – Ureteroscopy
4. 59.8 – Ureteral Catheterization
